# The Higher Education

**Published:** 1884-07

**Authors:** J. D. Moody

**Affiliations:** Dentist.


ARTICLE II

### THE HIGHER EDUCATION.

BY J. D. MOODY, DENTIST.

(Read before the Central Illinois Dental Society.)

We hear very much of late about "The High Education." Our dental journals have been full of it; the educational, theological and other professional periodicals literature, teem with it. We see it in our institutions of learning, by the gradual raising of their standard of examinations, both entrance and final. Our civil service agitation is giving a definite value to a knowledge qualification.

The unrest gendered by this struggle after higher intellectual attainment, is noticeable too, in the oft recurring discussions in our dental societies about the merit or demerit of our present standard of our collegiate instruction. At times the war of words wages hot and furious, passion is aroused; estrangement between the faculties of different colleges is brought about; plans for organized effort are frustrated, and individual effort is lost in the great sea of contention, and stands for naught. The reformers, who are always in advance of and who yet give shape to public sentiment, become disheartened, and the more timid ones prophesy failure. But notwithstanding these discouragements, we are advancing; the outpost of public opinion are, one by one, assailed; gained and held; and thus a progress is made which, though slight, is yet of a permanent character.

In this battle among the giants, where do we, as a profession, stand?

Whatever differences may exist between individual leaders, or what ever the clasping of dissonant views may do to retard, for the time being this tide of advancement, the fact nevertheless exists that the foremost men of our ranks demand, from the profession at large, that they adapt themselves to this higher educational standard.

By the world at large, we have not been accorded the scientific and intellectual position after which we have been striving; and perhaps for this reason, this state of unrest which promises future progress, is more apparent in our profession than in any other. From the gradual advance that is being made we have reason to hope that, sometimes in the future, ours will truly be one of the learned professions; and learned not in the sense that it *professes* a high standard, but in the fact that the rank and file *possess* this high standard.

Did I say we seem to be progressing towards this end? While I believe this to be a fact, and while we are told to possess our souls in patience, there is yet a dark side to the picture, which we cannot, without danger to our hoped for success, fail to look squarely in the face. And it is this that, while my foregoing statements are true of the front rank, the great masses of our profession have neither these attainments themselves, nor do they seem to care for them. This being the case reformers have a Herculean task before them.

Is this indifference real or seeming? Let us see first how it is with the educated portion of our ranks: A large per centage of the students in every graduating class, while being faithful, attentive and successful students, yet pursue their studies no farther than will warrant their obtaining their diplomas. Their every effort seems to be turned in this one direction of passing examinations and getting away. Work given to them, which while of value to them in after life, and counting nothing in examinations, is shirked. The advice of their superiors in these matters, is held in light esteem. They seem to be wholly absorbed with the ideas of getting away from study and to go to money-making. If in after life the study was resumed, it would, in a measure, atone for these short comings of student life But how few of them seriously pursue afterwards, either their dental studies or any line of investigation. Of course there are some who do go beyond the course actually required for the sake of the good it will do them in the future, but I believe these to be largely in the minority.

It may be objected, on the part of some, that in the time allotted to the course, only the attainments necessary for passing examinations can possibly be acquired. If this is so, justice to themselves, as well as to the profession they are about to enter, would require the student to go to college with a preparation which would enable him to make the most of the advantages offered. The same holds good with the medical and legal professions. We claim to be a learned profession; and yet our average dental student begins the study of his profession at the same mental point at which the ordinary college student begins but the preparation for the study of some profession. We have neither a preparatory course nor any assurance, hardly even a presumption, that our students will ever be learned men. Is this the fault of our colleges? I trow not. That they are trying to change the present condition of things, and compel a more highly educated class of applicants, is evidenced by the gradual raising of the standard of their entrance examinations. This trouble, of which I have been complaining, does not lie at the doors of our dental colleges. We find in it the seeming indifference, in the great mass of our profession, to the necessity of a higher grade of intellectual culture on the part of the dental student. The average applicant for a place as a dental student would not think of applying for a position with a Garretson, a Kingley, or a Boerdecker. The very consciousnsss of their ignorance would be too glaring to allow them to do it. Gentlemen, of course you recognize the converse of this proposition. The trouble lies within ourselves. Have I stated the case too strongly? The real fact is, that such a large number of the dentists, especially in the country districts, are such utter ignoramuses, as to give a corresponding tone and coloring to the dental profession at large. The little heaven from the colleges will be a long time in leaving the whole lump. I use the word "ignoramus," only in a professional and scientific sense. To so great an extent, that. in the eyes of the general public, we are professionally and scientifically of one caliber. There is but little discrimination on this point. Of course the dentist having real ability will, in time, have it recognized in his own circle; but the stranger coming to him eyes him askance, knowing full well that the mere title, "dentist," or "doctor," conveys with it no warrant of fitness. Indeed the public have been for so long a time educated in an opposite direction, that they are unfit even to place the proper estimate upon a man's value. Directly the opposite should be the case.

What then, in fact, are our professional attainments? While every part of our work has to do with the laws of physics, how many of us have the slightest conception of the molecular changes going on beneath our touch? How many of us seem to care? It has been years since we have known of the terrible dangers attending the growth and presence of micro-organisms, and yet how many dentists are there who can intelligently discuss a paper on that subject? While our success or failure depends, to so large an extent, upon knowing how to combat with microscopic things, how few of us use that instrument as one of our most valuable aids! While having to do with questions of life and death, we hardly know the first principles of biology. The chemical relations with which we have to do, confront us at every step, yet how many of us understand a chemical formula? While having to do with morbid pathology every day of our lives, how few of us can enter intelligently upon a rational explanation of these morbific growths, and consequently a rational treatment of the same.

A lecturer before a scientific body presents for our edification an excrescence growing out from a lizard's tail, and triumphantly claims it as a mistake of Nature, and we tender him a note of thanks to cover our inability to gainsay this travesty upon scientific teaching. We handle foils, alloys, plate, and tools, and yet the scientific of metallurgy is almost beyond our ken. We are dabbling in electricity, and yet how few of us can read with understanding a journal of electrical science? Now these thoughts force themselves upon our attention again and again. What are we going to do about it?

Gentlemen, I have presented this matter in this strong light to first arrest your attention, and secondly to bring your individual responsibility plainly before you. Having done so, may I point out two or three means by which we can and should, to our individual extent, correct in a measure this condition of things?

First, being in the profession throws upon you the obligation to do everything in your power to further its interests. You cannot be an intelligent man nor dentist, without extensive reading. It should be that to be known as a "dentist" would imply public recognition as a learned man. We should be looked up to in every community as authority in scientific matters. It properly belongs to us. Our profession should be a passport into every society. To accept anything short of this, is to lower our position from a profession to a mere trade. *The Scientific American*, *Science*, *Popular Science Monthly*, and kindred journals, should be part of our working force. But in our own line, how meagre is the array of dental works usually found in a dentist's office. You will generally find the dental bible and hymn book, (that's "Harris" and the "*Cosmos*.") and a chart of the 5th pair of nerves. Even of those who keep up with the current literature, but very few have a dental library worthy the name. The list of dental books is not a very large one. Let us begin by buying one—the one we most need perhaps—read it through carefully, not make a study of it, but carefully enought to know what you will and what you will not find in it. Then get another, do the same with it, master the contents if not the subject matter, and keep on doing so until you have persued every strictly dental work within reach. Include in the list an elementary work on Physiology, Histology, one on Morbid Anatomy, and one on the general practice of medicine. Having done so, and this is within the reach of all, you will be a mucn better dentist, and a much better citizen. Having gained a knowledge of the contents of your books, they will be referred to again and again, and will contribute much to your ultimate success. I know the dollars come hard, but they come harder when you begin to drop behind. I would urge this plan upon you all. It will place you upon a higher plane of intellectual life. It will compel respect. Now for another practical suggestion: I believe every professional man should have some hobby outside of the strict line of his work. It should be something upon which he could expend all his spare energy of body and mind, and something to which he could go for recreation. It should follow the bent of his mind, be either mechanical or chemical, in botany, zoology, entemolology, geology, electricity, or, in fact, anything, only so it will act as a support to the mental powers. Mere physical exercise, such as hunting, gymnastics; etc., is good in its place, but will not accomplish the good which the others will.

Our professional life throws a great strain upon the brain. Natural sleep is all the absolute rest that it needs. A change of brain effort, from bread winning to pastime, brings to light a marvellous provision of nature, to prevent either a stagnation or a total break down. Nature studies are the best. Wearied and perplexed with the cares of a day in the office, well nigh broken down, both in body and in mind, go out for an hour's butterfly hunt, or to look for some rare plant in which you believe to be hiding near by, and see how quickly all is changed. The tired muscles are forgotten—in fact are tired no longer—and refreshing sleep follows with its healing virtues. But to receive the most good from this by-play, no thought of gain must enter into it. It must not be made to contribute the value of one cent to your table, nor to your purse. It must be purely a recreation. Neither should this pastime be changed from time to time. All through life, the one hobby should be followed until you are a master in it. By doing this, at the end of your life you will have achieved great good to yourself, aud will have been able to contribute much to the sum total of human knowledge. Some of the best work ever done in special lines of research, has been accomplished in after office hours by men active in professional life. We have no right to be selfish. We owe something to society. The man who recognizes this obligation is in better condition, physically and socially in the end, than he does not. Now if these plans were brought to the attention of every one they might be accepted and acted upon with energy by a few, and in a half hearted manner by many more, but only to be dropped after a short trial from want of a stimulus to continued effort; or they might be carried on for a very long time by some, but in a sort of aimless manner that would defeat the very object to be gained by it.

The average man would not pursue, to as successful a conclusion, and study or work that had for its aim simply recreation or the general advancement of knowledge, as if something to act as a stimulus were held up before him.

In what manner shall we best provide this needed stimulus? May I offer a suggestion? Create a new dental degree, to be bestowed only upon such as have pursued a prescribed post-graduate course and passed satisfactory examinations in it. The examinations to be taken in one year or in twenty only insisting that they be taken. The post-graduate course to be, scientifically, a very broad one; perhaps insisting that it must bear, directly or indirectly, upon some phase of dental science. Also that the examination for this degree be preceded by one to show that the applicant is a more than ordinarily well informed person, or to have pursued some scientific study, *as* a study, or a pas time, and to have attained a degree of eminence in it, thus proving his right to belong to a learned profession. I would, in this way, create a sort of dental French academy, a dental academy of sciences, something which it would be an honor to belong to, something really worth striving for.

I use the word post-graduate simply for convenience and in a general sense, not confining it to those who have graduated, but applying it to a course of study outside of and beyond the ordinary collegiate course. The standard of this degree might be secured by law, through the inner-State Board of Examiners, or by inter-collegiate action. I am well aware that the creating of any new degree has been deprecated by many of our best men, and not without good reason. But let me for a moment examine the claims for my proposition. The safety of our profession and of the public alike, requires the dentist to have proper credentials before engaging in practice, showing that he has pursued a proper preparatory study. The easiest and best way to designate that is with the D. D. S. But, practically, that is *all* it means. The M. D. for us means no more, only that we can consult with a physician. Neither of these conveys any warrant that its possessor is a learned man, nor that he ever will be. Indeed, we know that the majority of both dental and medical graduates are but of mediocre calibre, and achieve but mediocre success. Of course, in any event, all men would not stand first. There always will be grades of intellect. But the ordinary man *can* occupy a much higher position than he now does. The great reason of so much mediocre talent is, that the stimulus which produced a successful examination was suddenly withdrawn, and the mind, clogged with care, was not able, unaided, to keep pace with its fellows in the intellectual race. Now place before the student, just as he has successfully passed his preparatory examination, another which even the brightest intellect must be years in attaining, but which we may all hopefully strive after, and you, at once, put a premium upon intellectual effort which will have a value for all time. It will be something worth working for; something which will unerringly testify that its possessor is a learned man.

A large proportion of the dentists in the country are in no sense learned men, and under present circumstances never will be. They came into the profession from various motives. Some came in by the college door, and many climed over in other ways. They all came in with hopes for the future, but with the majority, all energy and will-power have been crushed by disappointed hopes, and al projects of future college study blasted by want of funds. Time and money, both are wanting. Usually expenses cannot be thought of. The tread-mill of routine duty saps all vitality. No race-course has been marked out, no prize is within grasp. Stimulus of enthusiastic competitors is wanting; the mind becomes weary; the energies lag, and life becomes an aimless existence.

This is no fancy picture. It can be verified in a thousand instances. It may be objected, however, that the present race of unlearned dentists will, by virtue of our laws, die out in another generation, and thus a cure be affected of itself. While the condition of things would be bettered, it would be but in a slight degree, for I have shown the average graduate on entering upon his work, drops all but finger education and in no sense is a learned man. So it always will be. It is only by the untiring efforts of the few, who, endowed by nature with qualities of leadership, can by force of example or precept raise the standard of our profession, and, in a measure, keep the masses up to that standard. But in some such lives as I have indicated, the profession at large can be encouraged to work to a higher plane, and by diligence and perseverance win for themselves a place in the scientific world. We cannot afford to be left behind. Other professions are advancing, even the non-professional ranks are advancing. We, too, must advance or drop our pretensions.

The notes for this paper were prepared during the summer for this meeting. But since that time I found that in all probability the personel of the meeting would be different from what I had anticipated.

I have great sympathy for the country dentist, for I am one myself. Very largely he is the victim of unfortunate circumstances. A great many of them would be glad to go higher if they could. One great purpose of our society, in the first place, was to reach out after these, help them to better their condition, bring them into harmony with the spirit of progress, bring them into the State society and make them better men socially and professionally. They do not come into contact with the progressive ones. They have lost all vim, even care not to better themselves, thinking it beyond their grasp. And yet this very class give tone to our profession in the mind of the general public; and it was their friends who well-nigh defeated our present excellent law. I have come in contact with these a great deal, and I believe they can be reached. To such I offer the suggestions in this paper about personal effort. To others I offer the suggestion a new degree. It may bring a smile of ridicule, but I believe that within it there lies a germ capable of producing much of good to our profession. If there has been a practical paper in any of our journals on a course of reading, such as I have indicated, I have not seen it. I believe it is needed.

Gentlemen, let us catch hold ot our boot-straps and see if we cannot solve the problem.—*Ohio Journal of Dental Science.*

